# *Antrodia cinnamomea* Oligosaccharides Suppress Lipopolysaccharide-Induced Inflammation through Promoting *O*-GlcNAcylation and Repressing p38/Akt Phosphorylation

**DOI:** 10.3390/molecules23010051

**Published:** 2017-12-26

**Authors:** Junping Zheng, Siming Jiao, Qiongyu Li, Peiyuan Jia, Heng Yin, Xiaoming Zhao, Yuguang Du, Hongtao Liu

**Affiliations:** 1 Liaoning Provincial Key Laboratory of Carbohydrates, Dalian Institute of Chemical Physics Chinese Academy of Sciences, Dalian 116023, China; junpingzheng2013@163.com (J.Z.); yinheng@dicp.ac.cn (H.Y.); zhaoxm@dicp.ac.cn (X.Z.); 2 University of Chinese Academy of Sciences, Beijing 100049, China; 3 State Key Laboratory of Biochemical Engineering and Key Laboratory of Biopharmaceutical Production & Formulation Engineering, PLA, Institute of Process Engineering, Chinese Academy of Sciences, Beijing 100190, China; smjiao@ipe.ac.cn (S.J.); qiongyulee@hotmail.com (Q.L.); pyjia@ipe.ac.cn (P.J.); 4 Zhengzhou Institute of Emerging Industrial Technology, Zhengzhou 450000, China

**Keywords:** *Antrodia cinnamomea*, Akt, inflammation, *O*-GlcNAcylation, oligosaccharides

## Abstract

*Antrodia cinnamomea* (AC), an edible fungus growing in Taiwan, has various health benefits. This study was designed to examine the potential inhibitory effects of AC oligosaccharides on lipopolysaccharide (LPS)-induced inflammatory responses in vitro and in vivo. By trifluoroacetic acid degradation, two oligosaccharide products were prepared from AC polysaccharides at 90 °C (ACHO) or 25 °C (ACCO), which showed different oligosaccharide identities. Compared to ACCO, ACHO displayed better inhibitory effects on LPS-induced mRNA expression of pro-inflammatory cytokines including IL-6, IL-8, IL-1β, TNF-α and MCP-1 in macrophage cells. Further, ACHO significantly suppressed the inflammation in lung tissues of LPS-injected C57BL/6 mice. The potential anti-inflammatory molecular mechanism may be associated with the promotion of protein *O*-GlcNAcylation, which further skewed toward the marked suppression of p38 and Akt phosphorylation. Our results suggest that the suppressive effect of AC oligosaccharides on inflammation may be an effective approach for the prevention of inflammation-related diseases.

## 1. Introduction

*Antrodia cinnamomea* or *Antrodia camphorata* (AC), a special fungus that grows endemically in Taiwan, is traditionally used for functional foods and medicinal treatments. The raw extract from AC exhibits diverse pharmacological activities, including anti-inflammation, anti-tumor, anti-oxidation and hepatoprotection properties [1]. The AC extract is mainly composed of three categories of compounds: triterpenoids, dicarboxylic acids and polysaccharides [2]. Until now, the bioactivities of AC are mostly related to its lipophilic components such as ubiquinone derivatives, succinic acid derivatives and benzenoids [3,4,5,6], and compared to these chemicals, less attention has been paid to the polysaccharides from AC, which consists of a variety of monosaccharides such as galactose, glucose, mannose, arabinose, lyxose and fucose [7,8]. Limited studies have demonstrated the various bioactivities of AC polysaccharides: (1) preventing hepatic injury [9]; (2) alleviating inflammation [10]; (3) reducing oxidative stress [11]; (4) modulating the immune system [12]; (5) helping inhibit tumor proliferation [13]. However, the molecular mechanism of the biological functions of AC polysaccharides, with regard to its regulation of the post-transcriptional modification of proteins or other signaling pathways, remains poorly understood.

*O*-linkage of *N*-acetylglucosamine (*O*-GlcNAc) to serine/threonine residues of target proteins is termed as *O*-GlcNAc modification (*O*-GlcNAcylation) [14], which is a novel post-translational modification attached by *O*-GlcNAc transferase (OGT) [15] and removed by *O*-GlcNAcase (OGA) [16]. Since the substrate of OGT, uridine diphosphate *N*-acetylglucosamine (UDP-GlcNAc), is derived from the metabolism of glucose by the hexosamine biosynthetic pathway, *O*-GlcNAcylation is regarded as a nutrient sensor reflecting a healthy status in vivo [17]. Also, by competing for the same modification sites as phosphorylation, *O*-GlcNAcylation is found to influence multiple biological processes such as transcription, translation, signal transduction, cell cycle and protein degradation [18]. So far, *O*-GlcNAcylation has been proven to be involved in several diseases, including diabetes, neurodegenerative diseases, cancers and inflammation, etc. [19,20].

Several pathological factors such as hemorrhage injury and infection, induce acute inflammatory responses accompanied by tissue damage. Series of evidence indicate the significance of *O*-GlcNAcylation in inflammatory responses, but the exact regulatory mechanism of *O*-GlcNAc modification has not been clarified due to the complex homeostasis until now. In some cases, it seems that inhibition of protein *O*-GlcNAcylation alleviated the occurrence of inflammation [19,20]. There are other studies demonstrating the down-regulation of protein *O*-GlcNAcylation under inflammatory status, while the increased *O*-GlcNAc level on proteins exerts anti-inflammatory properties and reduces organ damage [21,22]. These contradictory conclusions suggest that it is necessary to gain further insight into the pivotal role of *O*-GlcNAcylation in inflammatory reactions.

In contrast to polysaccharides, some oligosaccharides with lower degrees of polymerization usually display better solubility in water, indicating a higher medical application potential [23]. So far, there is no report about the preparation or activity of AC oligosaccharides. We hypothesized that AC oligosaccharides may exert an anti-inflammatory effect by modulating protein *O*-GlcNAcylation. Therefore, we aimed to examine whether AC oligosaccharides suppress the inflammatory responses, and if so, by what mechanism. In the present study, we aimed to assess the potential inhibitory effects of AC oligosaccharides on lipopolysaccharide (LPS)-induced inflammation in vitro and in vivo. We also wanted to investigate the roles of *O*-GlcNAcylation, mitogen-activated protein kinases (MAPKs) and Akt in the anti-inflammatory process initiated by AC oligosaccharides.

## 2. Results

### 2.1. Composition Analysis of ACCO and ACHO 

**Figure 1:**
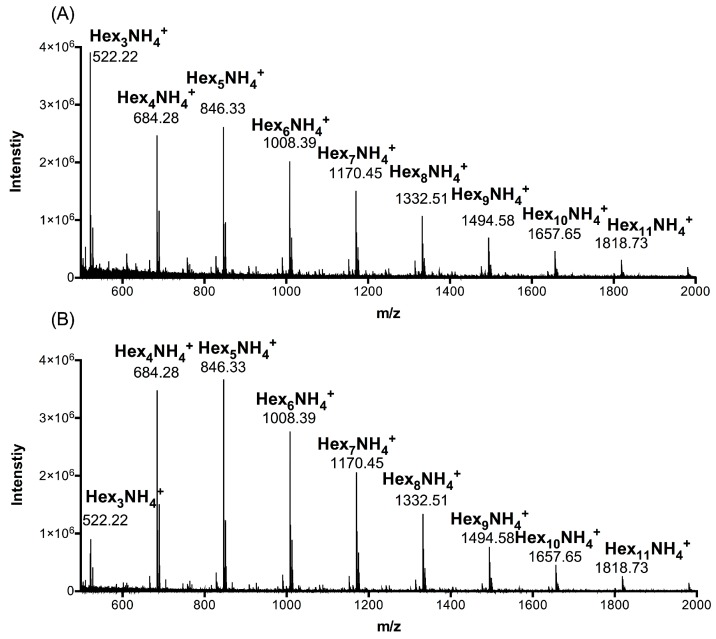
UPLC-MS analysis of *Antrodia cinnamomea* (AC) polysaccharides at 90 °C (ACHO) or 25 °C (ACCO). (**A**) Polymerization degree identification of ACCO; (**B**) Polymerization degree identification of ACHO. Hex represents hexose.

To determine the distribution of polymerization degree of AC polysaccharides at 90 °C (ACHO) or 25 °C (ACCO), we carried out UPLC-MS analyses. As shown in Figure 1, the ion peaks at *m*/*z* 522.22, 684.28, 846.33, 1008.39, 1170.45, 1332.51, 1494.58, 1657.65 and 1818.73 separately represented the oligosaccharide constituents of ACCO (Figure 1a) or ACHO (Figure 1b), which were oligomeric forms of hexose and the polymerization degree was 3–11. According to the ultra-performance liquid chromatography (UPLC) of ACCO and ACHO (Figures S1 and S2), their weight percentages in the oligo mixtures are presented in Table 1.

We further analyzed the monosaccharide identity of ACCO and ACHO using capillary electrophoresis, which suggested that both oligosaccharides were composed of five monosaccharides, namely mannose, galactose, glucose, fucose and arabinose (Figure S3). The relative concentration and percentage of each monosaccharide was quantified in Table 2, and the results show that ACCO or ACHO displayed similar contents of glucose, galactose and mannose. However, different levels of fucose (0.53% for ACHO, 0.17% for ACCO) and arabinose (0.45% for ACHO, 1.18% for ACCO) were observed between the two oligosaccharides.

### 2.2. ACCO and ACHO Suppressed LPS-Induced Inflammation in Macrophage Cells

**Figure 2:**
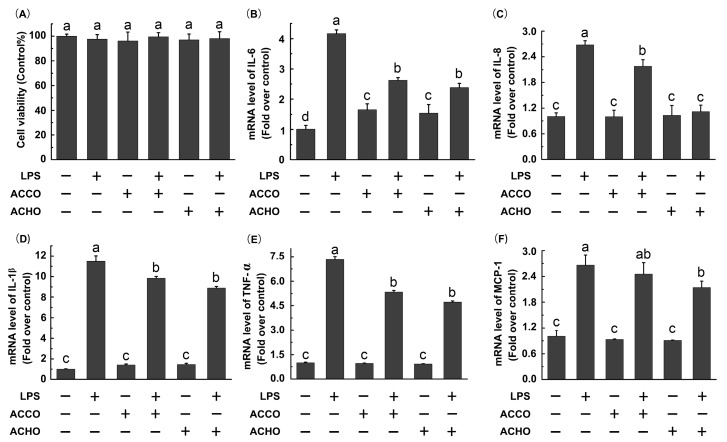
Effects of ACCO and ACHO on viability and inflammatory reaction in lipopolysaccharide (LPS)-stimulated macrophage cells. (**A**) RAW264.7 cells were treated with LPS (200 ng/mL), ACCO (100 µg/mL) or ACHO (100 µg/mL) for 24 h. After that, the cell viability was tested by a MTT assay; (**B**–**F**) RAW264.7 cells were pre-treated with ACCO (100 µg/mL) or ACHO (100 µg/mL) for 12 h and then exposed to LPS (200 ng/mL) for 6 h. Next, the mRNA levels of IL-6 (**B**), IL-8 (**C**), IL-1β (**D**), TNFα (**E**) and MCP-1 (**F**) were determined by RT-PCR analysis. Data are the mean ± SD (*n* = 3). Data with different superscript letters are significantly different (*p* < 0.05).

To decide whether LPS or AC oligosaccharides altered the viability of RAW264.7 macrophage cells, a MTT assay was performed. Cells were treated with LPS (200 ng/mL), ACCO (100 μg/mL) or ACHO (100 μg/mL) for 24 h. As shown in Figure 2A, there was no significant difference of viability among all groups at the studied concentrations. Next, we tested the potential inhibitory effects of ACCO and ACHO on LPS-induced inflammation. The result indicates that mRNA levels of IL-6, IL-8, IL-1β, TNF-α and MCP-1 were significantly increased after LPS stimulation for 6 h (*p* < 0.05 vs. control group) by RT-PCR analysis (Figure 2B–F). Furthermore, the up-regulated transcription of above pro-inflammatory cytokines were dramatically suppressed by ACCO or ACHO pretreatment for 12 h (*p* < 0.05 vs. LPS group). Interestingly, it seemed that ACHO displayed better anti-inflammatory effects on LPS than that of ACCO by trends. Therefore, we focused our studies on ACHO in the next experiments.

### 2.3. ACHO Inhibited the Phosphorylation of p38 and Akt in LPS-Induced Macrophage Cells

**Figure 3:**
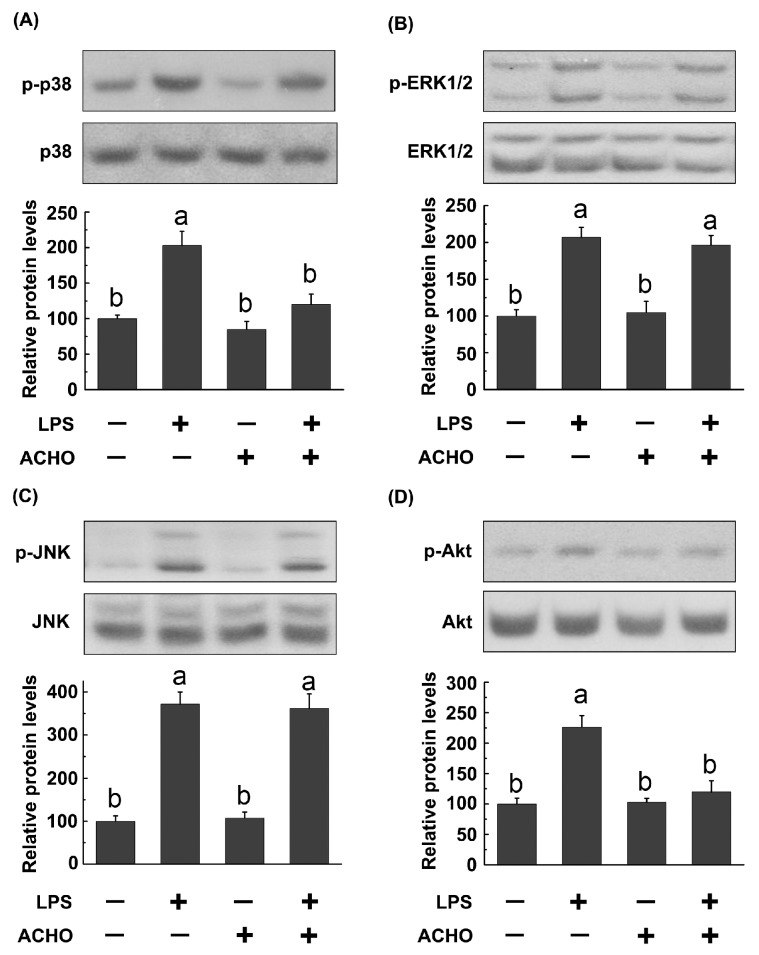
Inhibitory effect of ACHO on activation of MAPK and Akt signaling pathways in LPS-induced macrophage cells. RAW264.7 cells were pretreated with ACHO (100 µg/mL) in FBS-free medium for 12 h and then exposed to LPS (200 ng/mL) for 30 min. After that, cells were collected and the phosphorylated levels of p38 (**A**); ERK1/2 (**B**); JNK (**C**) and Akt (**D**) were determined by western blot. Data represent the mean ± SD (*n* = 3). Data with different superscript letters are significantly different (*p* < 0.05).

To explore the potential molecular mechanisms by which ACHO displayed the anti-inflammatory activity, RAW264.7 macrophage cells were pretreated with ACHO (100 μg/mL) in fetal bovine serum (FBS)-free medium for 12 h and then exposed to LPS (200 ng/mL) for 30 min. After that, the activation of MAPK and Akt signaling pathways was monitored by western blot. As indicated in Figure 3, LPS stimulation led to remarkably increase in the phosphorylation of p38, ERK1/2, JNK and Akt (*p* < 0.05 vs. control group). Interestingly, ACHO pretreatment significantly down-regulated the levels of p-p38 and p-Akt in LPS-induced macrophage cells (*p* < 0.05 vs. LPS group) (Figure 3A–D), but had no effect on the activation of p-ERK1/2 and p-JNK.

### 2.4. ACHO Reversed Down-Regulation of O-GlcNAcylation in LPS-Induced Macrophage Cells

**Figure 4:**
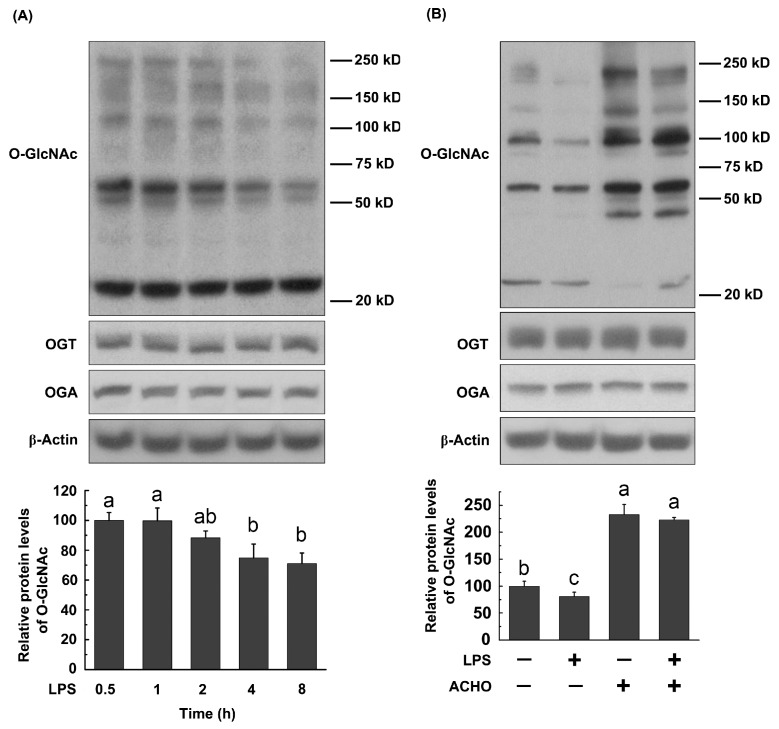
Effects of LPS and ACHO on protein *O*-GlcNAcylation in macrophage cells. (**A**) RAW264.7 cells were treated with LPS (200 ng/mL) for 0–8 h; (**B**) RAW264.7 cells were pretreated with ACHO (100 µg/mL) for 12 h and then exposed to LPS (200 ng/mL) for 8 h. After that, cells were collected and analyzed by western blot. Data are represented as the mean ± SD (*n* = 3). Data with different superscript letters are significantly different (*p* < 0.05).

Since *O*-GlcNAc modification has been proven to play an important role in inflammatory processes [24], we detected the *O*-GlcNAc levels in macrophage cells treated with LPS, ACHO or both. The results show that total *O*-GlcNAc levels in RAW264.7 cells were decreased by LPS (200 ng/mL) in a time-dependent manner (*p* < 0.05 vs. control group, Figure 4A), which was totally reversed by ACHO pretreatment for 12 h (100 µg/mL, *p* < 0.05 vs. LPS group, Figure 4B). Noticeably, ACHO alone markedly promoted the protein *O*-GlcNAcylation in macrophage cells (*p* < 0.05 vs. control group). Neither LPS nor ACHO affected the protein levels of OGT and OGA (Figure 4A,B).

### 2.5. ACHO Inhibited p38 and Akt Phosphorylation by Promoting Protein O-GlcNAcylation in LPS-Induced Macrophage Cells

**Figure 5:**
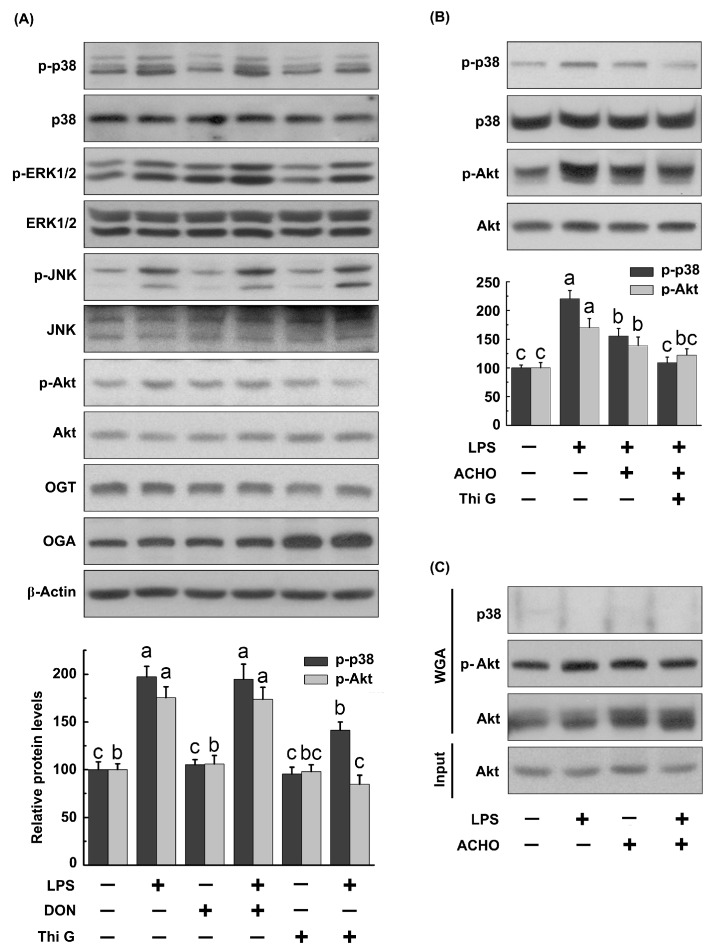
Suppression of MAPK and Akt phosphorylation by protein *O*-GlcNAcylation in LPS-induced macrophage cells. (**A**,**B**) RAW264.7 cells were pretreated with Thi G (50 nM), 6-diazo-5-oxo-l-norleucine (DON) (5 μM) or ACHO (100 μg/mL) in FBS-free medium for 12 h and then exposed to LPS (200 ng/mL) for 30 min. After that, cells were collected, and the protein samples were analyzed by western blot; (**C**) RAW264.7 cells were pretreated with ACHO (100 μg/mL) in FBS-free medium for 12 h and then exposed to LPS (200 ng/mL) for 30 min. Then, the *O*-GlcNAcylated proteins were pulled down by WGA-agarose and captured using the antibodies against p38, Akt or p-Akt. Data represent the mean ± SD (*n* = 3). Data with different superscript letters are significantly different (*p* < 0.05).

To delineate the role of *O*-GlcNAc in LPS-induced activation of MAPKs and Akt, RAW264.7 cells were pretreated with Thiamet G (50 nM, a potent inhibitor of OGA) to raise *O*-GlcNAc levels or with 6-diazo-5-oxo-l-norleucine (DON) (5 μM) to lower *O*-GlcNAc levels for 12 h, and then exposed to LPS (200 ng/mL) for 30 min. DON can reduce *O*-GlcNAc levels due to its ability to inhibit glutamine fructose-6-amidotransferase. As displayed in Figure 5A, Thi G significantly suppressed LPS-induced up-regulation of p-p38 and p-Akt in macrophage cells (*p* < 0.05 vs. LPS group), while DON did not. Additionally, both Thi G and DON failed to reverse the increase in p-ERK1/2 and p-JNK by LPS stimulation. Moreover, an accumulated suppressive effect on p-p38 and p-Akt was observed in cells pretreated with ACHO plus Thi G before LPS explosion (Figure 5B).

Considering the possible interplay at same modification sites between *O*-GlcNAcylation and phosphorylation, we determined the effect of ACHO on *O*-GlcNAc levels of p38 and Akt in LPS-induced macrophage cells. The cellular *O*-GlcNAcylated proteins were pulled down by WGA-agarose and further captured using the antibodies against p38, Akt or p-Akt (Thr 308). Regretfully, no *O*-GlcNAcylated p38 was detected in this study. Though ACHO raised the *O*-GlcNAc level of total Akt, it had no effect on the *O*-GlcNAcylation of p-Akt in LPS-induced cells (Figure 5C).

### 2.6. ACHO Suppressed the Inflammatory Reaction in Lung Tissues of LPS-Injected Mice

**Figure 6:**
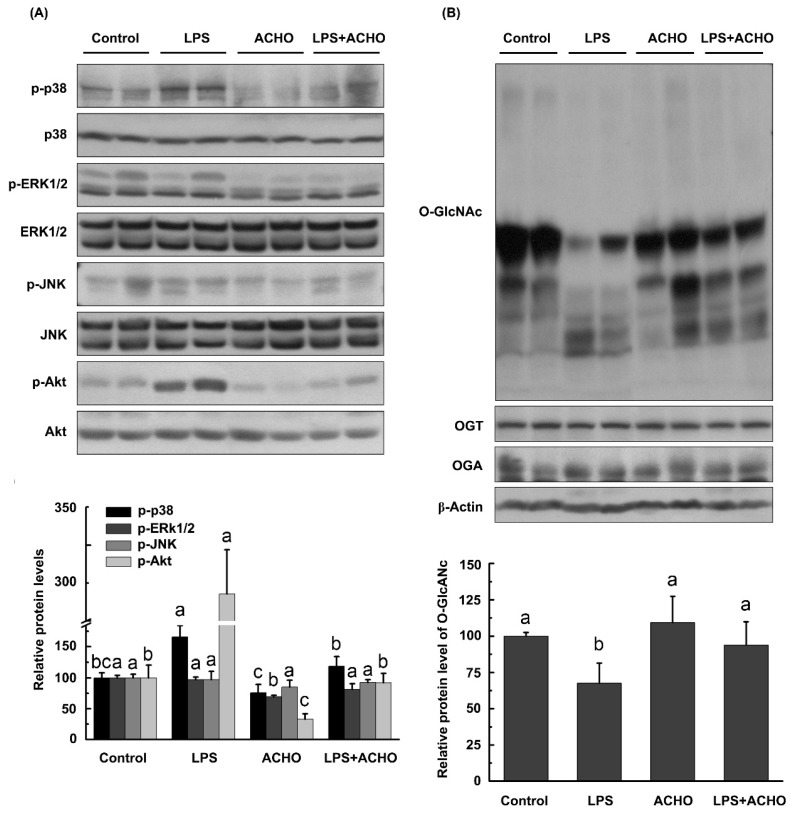
Effect of ACHO on MAPKs, Akt and protein *O*-GlcNAcylation in the lung tissues of LPS-injected mice. (**A**) Suppression of ACHO on the activation of MAPKs and Akt in lung tissues after LPS injection; (**B**) Reversal of ACHO on the decrease in protein *O*-GlcNAcylation in lung tissues after LPS injection. C57BL/6 mice were pre-treated with ACHO (1 mg/mL in drinking water) for two weeks followed by LPS injection (3 mg/kg) intraperitoneally for 24 h. After that, mice were euthanized, and lung tissues were collected for western blot assay. Data represent the mean ± SD (*n* = 5). Data with different superscript letters are significantly different (*p* < 0.05).

We further investigated whether ACHO inhibited LPS-induced acute inflammation in vivo. C57BL/6 mice were pre-treated with ACHO (1 mg/mL in drinking water) for two weeks followed by injection of LPS (3 mg/kg) for 24 h. The results show that there was no significant difference in body weight between all groups on the day of tissue collection (data not shown). Compared with control group, the lung tissues of LPS-injected mice had substantial increase in the phosphorylation of p38 and Akt (*p* < 0.05) and a considerable decrease in the protein *O*-GlcNAcylation (*p* < 0.05), both of which were almost entirely reversed by ACHO administration (*p* < 0.05 vs. control group) (Figure 6A,B), in accordance with the in vitro study. Also, ACHO administration failed to reduce the phosphorylated levels of ERK1/2 and JNK (Figure 6A). Even though OGT and OGA are the only two enzymes responsible for protein *O*-GlcNAcylation, it seemed that the production of both enzymes was not affected by either LPS injection or ACHO administration.

**Figure 7:**
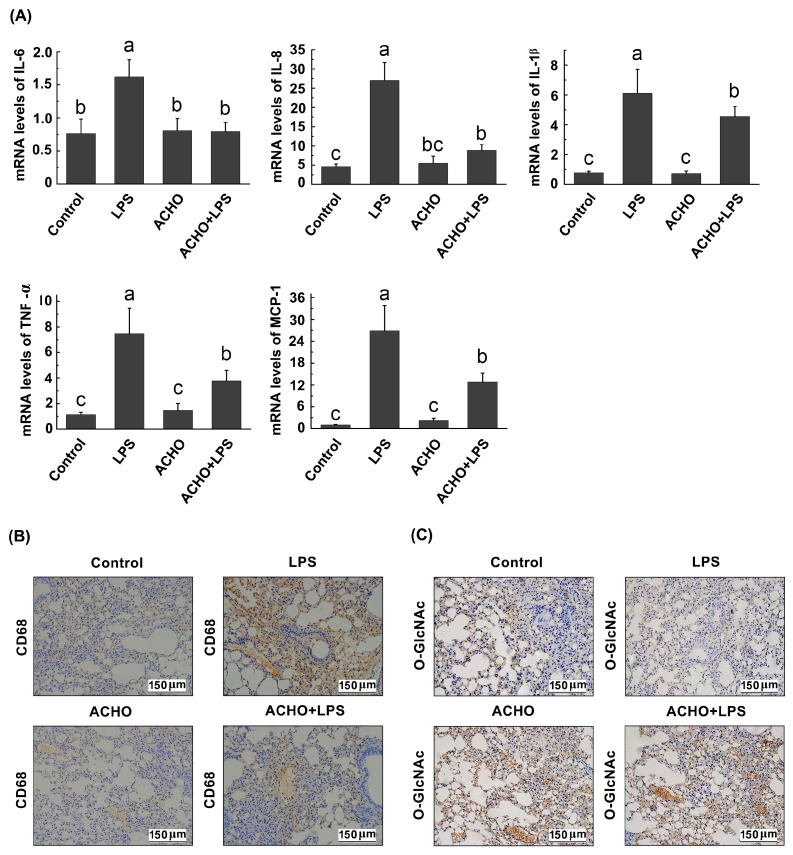
Effect of ACHO on transcriptional activation of pro-inflammatory cytokines (**A**), macrophage infiltration (**B**) and protein *O*-GlcNAcylation (**C**) in lung tissues of LPS-injected mice. C57BL/6 mice were pre-treated with ACHO (1 mg/mL in drinking water) for two weeks followed by LPS (3 mg/kg) injection intraperitoneally for 24 h. After that, mice were euthanized, and lung tissues were collected for RT-PCR assay or immunochemistry assay. (**A**) Expression of pro-inflammatory cytokines at mRNA level in lung tissues of LPS-injected mice, including IL-6, IL-8, IL-1β, TNF-α and MCP-1; (**B**) Effect of ACHO on CD68 (cluster of differentiation 68) production in lung tissues of LPS-injected mice (×200); (**C**) Effect of ACHO on protein *O*-GlcNAcylation in lung tissues of LPS-injected mice (×200). Data represent the mean ± SD (*n* = 5). Data with different superscript letters are significantly different (*p* < 0.05).

To assess anti-inflammatory activities of ACHO, the transcription levels of pro-inflammatory cytokines were examined in lung tissues (Figure 7A). The result indicates that LPS injection significantly increased the expression of pro-inflammatory cytokines at mRNA level, including IL-6, IL-8, IL-1β, TNF-α and MCP-1 (*p* < 0.01 vs. control group). On the contrary, ACHO remarkably reversed LPS-induced over-expression of above cytokines (*p* < 0.05 vs. LPS group), suggesting the strong suppressive effect of ACHO on acute inflammatory response.

Finally, the immunohistochemical examination was performed to further confirm the inhibitory effect of ACHO on inflammatory damage to lung tissues of LPS-injected mice. The morphological staining provided clear evidence that lung tissues of LPS-injected mice had an evident increase in the expression of CD68, a molecular marker of macrophages (Figure 7B). In contrast, ACHO administration significantly ameliorated the infiltration of macrophage cells, which could be seen from the reduced CD68 level. In addition, ACHO administration reversed the reduction of protein *O*-GlcNAcylation in lung tissues of mice with LPS injection (Figure 7C).

## 3. Discussion

Due to the evident therapeutic activity of AC against hyperlipidemia, liver disorders, cancers, inflammation and other diseases, the studies on its pharmacological effect have attracted much attention in recent years. As a major component of AC extract, the AC polysaccharides and their related derivatives deserve to be deeply investigated for the future medicinal application. In this study, for the first time we prepared AC oligosaccharides and demonstrated that ACHO effectively attenuated the inflammatory responses to LPS both in vitro and in vivo.

Because of the distinct water solubility and viscosity of polysaccharides, the polysaccharide yield of plant or fungi differs under different extraction temperatures. Therefore, the constitutive polysaccharides extracted by cold and hot water are usually different [25]. Noticeably, most of AC polysaccharides were extracted using hot water [6,9,10,11]. So far, no studies compared the anti-inflammatory effects of AC oligosaccharides with different extraction temperatures. Consistent with a previous study on AC polysaccharides [6], we found that mannose, galactose, glucose, fucose and arabinose were the building blocks of ACCO and ACHO (Table 2). Interestingly, ACHO displayed a better suppressive effect on LPS-induced inflammation than that of ACCO (Figure 2), in line with the report that polysaccharides extracted by cold and hot water exerted different bioactivities [26]. This may have contributed to their distinctly constitutive abundance of fucose and arabinose, or the difference in relative abundance of trisaccahride, tetrasaccharide, pentasaccharide and hexa-saccharide (Table 1 and Table 2). Indeed, the evidence shows that fucoidan (high fucose proportion) had potent inhibitory effect on the inflammation [27]. Due to the low abundance of arabinose and fucose, both failed to be detected by mass spectrum analysis. More detailed structural information about ACHO and ACCO should be elucidated in future studies.

As an inflammatory mediator, IL-8 causes immune cells to migrate to the site of infection and plays a key role in host defense against pathogens such as LPS. MCP-1 is an intercellular adhesion molecule, which is responsible for the activation of monocytes and macrophages. Also, IL-6, IL-1β and TNF-α are pro-inflammatory cytokines involved in LPS-induced inflammation. In this study, all of the above cytokines were activated in RAW 264.7 macrophage cells by LPS stimulation. On the other hand, both ACHO and ACCO displayed statistically suppressive effects on the over-expression of these cytokines, indicating a great potential of both AC oligosaccharides in acute infection diseases by in vitro pathogens.

Considering that LPS-induced inflammatory responses are usually mediated by MAPKs and Akt, we next focused our study on the two pathways. MAPK subfamily members, consisting of p38, ERK1/2 and JNK, play critical roles in activating host immune responses and are a frequent target of pathogenic effectors [28]. Akt, also called PKB, is a key player in cellular processes such as cell proliferation, apoptosis, inflammation and other signaling pathways [29]. It was reported that ethanol extracts of AC exhibited cancer cell migration through the MAPKs and PI3K/Akt pathway [30,31]. The activation of NF-κB and MAPK was also regulated by methanol extract of AC [5]. In the present study, ACHO (100 µg/mL) strikingly suppressed the LPS-induced up-regulation of p-p38, p-Akt and pro-inflammatory cytokines in both macrophage cells and mouse lung tissues (Figure 3, Figure 6A and Figure 7A), in agreement with previous studies about AC polysaccharides [32]. Collectively, the reduced phosphorylation of p38 and Akt after ACHO treatment in vitro and in vivo may have prevented the transcription activation of the above pro-inflammatory cytokines.

**Figure 8:**
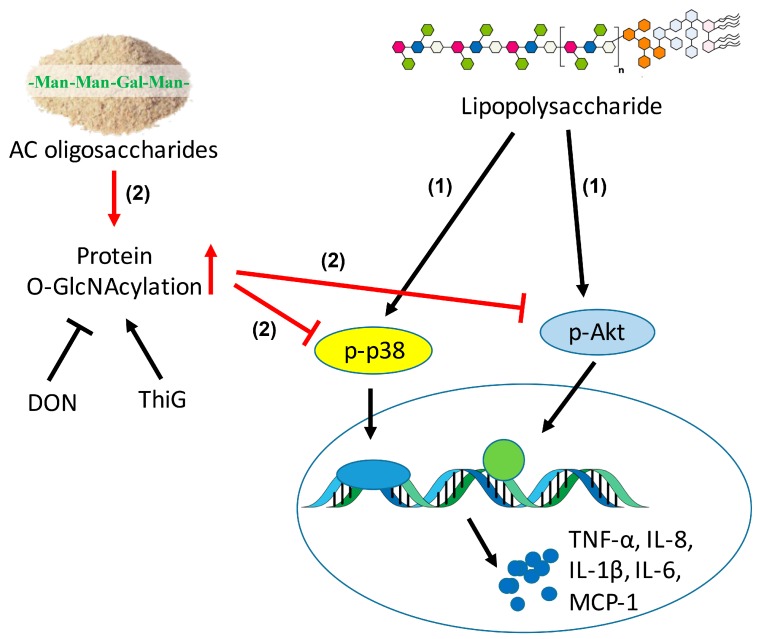
The schematic diagram that ACHO inhibited LPS-induced inflammation. (1) LPS induced the over-expression of IL-6, IL-8, IL-1β, TNF-α and MCP-1 at mRNA levels by up-regulation of p-p38 and p-Akt; (2) ACHO promoted the protein *O*-GlcNAcylation, which further suppressed phosphorylation of p38 and Akt and subsequently inhibited LPS-mediated inflammation.

As an important post-translational modification, *O*-GlcNAcylation modulates the function of more than 1000 cytoplasmic or nuclear proteins, is involved in the regulation of signal transduction and influences a wide range of cellular processes [33]. As for the role of *O*-GlcNAcylation in activation of p38 and Akt, contradictory conclusions were reached in different study groups. For example, it has been suggested that the increase of protein *O*-GlcNAcylation supported p38 and Akt activation [34,35]. Taking a different view point, Fulop et al. demonstrated that increased *O*-GlcNAc modification was negatively related to p38 activation [36]. The discrepancy among previous studies could be due to the different cell types or tissues under various experimental conditions. Here we found that the elevation of *O*-GlcNAcylation by Thi G (an inhibitor of OGA) effectively blunted the phosphorylation of p38 and Akt in LPS-induced macrophage cells, and such suppressive effect was strengthened by addition of ACHO (Figure 5). This was further supported by the reduced macrophage infiltration and increased protein *O*-GlcNAcylation in the lung tissues of LPS-injected mice with ACHO administration (Figure 7B,C). It is thus possible that ACHO inhibited p38 and Akt activation through the promotion of protein *O*-GlcNAcylation (Figure 8).

The *O*-GlcNAcylation may regulate phosphorylation by competing for the same amino acid or affect the neighboring sites [33]. Early studies showed that there were multiple modification sites of *O*-GlcNAcylation on Akt [35,37,38]. Based on the above, we presumed that ACHO may increase the protein *O*-GlcNAcylation, which competed for the same modification sites on p38 and Akt with phosphorylation. Regretfully, no *O*-GlcNAc modification was found on p38 kinase in our experiment. Also, we found no changes of *O*-GlcNAcylation at Thr 308 (Figure 5C) and Ser 473 (data not shown) residues on p-Akt, even though the phosphorylated Akt level was decreased after ACHO pre-treatment in LPS-induced macrophage cells. Therefore, it was plausible that ACHO suppresses LPS-induced inflammation by increasing the *O*-GlcNAcylation of regulators up-stream of p38 and Akt, and this will be further investigated in our future work.

## 4. Materials and Methods

### 4.1. Reagents

In this study, the antibodies respectively against p38, p-p38, ERK, p-ERK, Akt1/2/3, p-Akt1/2/3, OGT, OGA, CD68 and β-actin were purchased from Santa Cruz Biotechnology (Santa Cruz, CA, USA). Monoclonal Anti-β-*O*-Linked *N*-Acetylglucosamine Clone CTD110.6 was obtained from Sigma (St. Louis, MO, USA). Other antibodies including anti-JNK, anti-p-JNK, Horseradish peroxidase (HRP)-conjugated goat anti-rabbit IgG and HRP-conjugated goat anti-mouse IgG were purchased from Cell Signaling Technology (Danvers, MA, USA).

Chemicals such as 1-phenyl-3-methyl-5-pyrazolone (PMP), 3-(4,5-dimethylthiazol-2-yl)-2,5-diphenyltetrazolium bromide (MTT), Tris, Tween 20, trifluoroacetic acid, LPS (from *Escherichia coli* 055:B5), 6-diazo-5-oxo-L-norleucine (DON) and bovine serum albumin were obtained from Sigma. The ABC kit and Agarose wheat germ agglutinin (WGA) were purchased from Vector Laboratories (Lowellville, OH, USA). Thiamet G (Thi G) was obtained from Selleck Chemicals (Houston, TX, USA). RPMI 1640 was purchased from Corning Incorporated (Corning, NY, USA), penicillin, streptomycin and heat-inactivated fetal bovine serum (FBS) was from Gibco (Grand Island, NY, USA). All the other chemicals including sodium dodecyl sulfonate, ammonium persulfate, isopropanol, hydrochloric acid, glycine, sodium chloride and ammonia were obtained from Sinopharm Chemical Reagent Co., Ltd. (Shanghai, China).

### 4.2. Preparation of Oligosaccharides from AC Fruit Bodies

The fruit bodies of AC were provided by Wellhead Biological Technology Corp (Taiwan, China) in this study. The *Antrodia cinnamomea* (AC) was cultured on woods of *Cinnamomum kanehirai* under controlled environment. Firstly, the water extract was prepared by mixing 20 g of the dried fruit bodies powder in 800 mL of distilled water before agitation at 200 rpm for 4 h at 25 °C or 90 °C. The solution was cooled to room temperature (22 °C) followed by centrifugation at 4000× *g* for 30 min. After that, the solution of crude polysaccharides was filtered using a SHZ-3 Rotavapor vacuum concentrator (Yikai Co., Ltd., Shanghai, China) at 40 °C to obtain a final volume of 100 mL. Then, 300 mL of 95% ethanol (*v*/*v*) was added and incubated for 18 h. Following by the centrifugation at 4000× *g* for 30 min, the precipitated crude polysaccharide was re-dissolved in H_2_O. Next, the mixture of chloroform and *n*-Butanol (3:1, *v*/*v*) was added to remove the protein from crude polysaccharide as reported before [39]. After lyophilization, the polysaccharide residue was stored at −80 °C.

For the oligosaccharide preparation, 1 g of above AC polysaccharide extracted at 25 °C or 90 °C was dissolved in 100 mL of H_2_O, and then mixed with 1.25 M of trifluoroacetic acid (TFA, 1:9, *v*/*v*). Under the N2 atmosphere, polysaccharide degradation was initiated at 95 °C for 1.5 h. After the reaction, ammonia (1:10, *v*/*v*) was added for the neutralization. Finally, two oligosaccharide products were separately prepared: (1) ACCO, from AC polysaccharide extracted at 25 °C; (2) ACHO, from AC polysaccharide extracted at 90 °C. Furthermore, the monosaccharide composition was determined by capillary electrophoresis analysis (Figure S3).

### 4.3. Ultra Performance Liquid Chromatography-Mass Spectrometry (UPLC-MS) Analysis of ACCO and ACHO

For the composition analysis and molecular weight determination of ACCO and ACHO, UPLC-Q-TOF-MS analysis was conducted using a Waters ACQUITY UPLC system (Waters Corp., Milford, MA, USA). The analysis was performed on an Acquity UPLC BEH Amide column (2.1 mm × 150 mm; 1.7 μm, Waters Corporation) at a flow rate of 0.3 mL/min by gradient elution using 100 mM ammonium formate in water (A) and acetonitrile (B) at a flow rate of 0.3 mL/min. The gradient profile was: 0–2 min (A: 15–15%), 2–25 min (A: 15–40%), 25–40 min (A: 40–50%), 40–41 min (A: 50–80%), and held for 5 min. The gradient was recycled back to 15% in 1 min, and held for 3 min for the next run. The injection volume was 10 μL. The column temperature was set to 40 °C.

Mass spectrometry was carried out using a Waters Synapt™ mass spectrometer (Waters Corp.). Ionization was performed in the negative electrospray (ESI) mode. The MS parameters were as follows: capillary voltage, 3.6 kV; cone voltage, 20 V; source temperature, 150 °C; desolvation temperature, 350 °C; gas flows of cone and desolvation, 40 and 800 L/h, respectively. The data were processed using MassLynx™ 4.1 software (Waters Corp.). The oligosaccharide profiles were acquired over a mass range of *m*/*z* 500–2000.

### 4.4. Cell Culture

The RAW264.7 murine macrophage cells were from American Type Culture Collection (ATCC, Manassas, VA, USA) and cultured in RPMI 1640 medium containing 10% FBS, penicillin (100 U/mL) and streptomycin (100 μg/mL). Cells were incubated at 37 °C in a humidified atmosphere of 5% CO_2_. The stocking solutions of ACCO (10 mg/mL), ACHO (10 mg/mL) and LPS (20 µg/mL) were dissolved in PBS. Before used for cellular experiments, the above stockings were further diluted in culture medium to final concentrations.

For cell viability assay, the RAW264.7 cells were seeded into 96-well plates at density of 1 × 10^4^ cells/well. After 12 h culture, cells were treated with LPS (200 ng/mL), ACCO (100 µg/mL) or ACHO (100 µg/mL) for 24 h. The MTT colorimetric assay was performed as previously reported [40]. For other analysis, the macrophage cells were seeded into six-well plates at density of 4 × 10^5^ cells/well. After 12 h culture, cells were pre-treated with PBS, ACCO (100 µg/mL) or ACHO (100 µg/mL) for 12 h and then exposed to LPS (200 ng/mL) for 0–8 h. After that, cells were washed twice with ice-cold PBS and scraped for lysis using RIPA buffer. After centrifugation, the supernatant was collected for western blotting. For RNA extraction, cells were washed, and total RNA were extracted by using a commercialized kit.

### 4.5. Animal Experiment

Male C57BL/6 mice (20 ± 2 g, six weeks old) were purchased from the model animal research center of Nanjing University (Nanjing, China). Mice were housed in a controlled environment with a 12 h light-dark cycle at 25 ± 2 °C and had free access to food and water. After a week, they were randomly divided into four groups (*n* = 5), and every group of mice were housed individually. The group information was as follows: control group; LPS group; ACHO group; ACHO+LPS group. Mice were treated with normal drinking water or ACHO (1 g/L in drinking water, at the dose of around 200 mg/kg/day) for two weeks, followed by injection of PBS or LPS (3 mg/kg) from *E. coli* intraperitoneally. After that for 24 h, all mice were euthanized, and the lung tissues were collected and stored at −80 °C. The procedures of animal experiments were approved by the Animal Ethical Experimentation Committee of Institute of Process Engineering, Chinese Academy of Sciences (Beijing, China) and in accordance with the National Act on Use of Experimental Animals (permission number: SYXK2014-0002, China).

### 4.6. Isolation and Analysis of O-GlcNAcylated Proteins

The enrichment of *O*-GlcNAcylated proteins was performed as previously described with some modifications [41]. In brief, cell lysates (200 μg) were incubated with 30 μL of Agarose bound Wheat Germ Agglutinin (1:1, *v*/*v* slurry) and mixed by rotation for 2 h at 4 °C. Next, the beads were pelleted and washed with Cell Lysis Buffer (Cell Signaling Technology) three times. The attached proteins were eluted with SDS-PAGE sample buffer, separated by SDS-PAGE and analyzed by western blot using the antibodies against p38, Akt, or p-Akt.

### 4.7. Western Blotting

The total proteins of macrophage cells or lung tissues were homogenized by RIPA buffer (Cell Signaling, Danvers, MA, USA) supplemented with protease inhibitor Cocktail (Merck, Darmstadt, Germany). The protein concentration was determined using a BCA Protein Assay Kit (Beyotime, Shanghai, China). Proteins were separated on 8–12% SDS-PAGE and transferred to the polyvinylidene fluoride (PVDF) membranes. Membranes were blocked with 5% non-fat milk for 1 h at room temperature in TBST buffer (Tris 10 mM, NaCl 150 mM, pH 7.6, 0.1% Tween 20) and probed with various primary antibodies overnight at 4 °C. Then, membranes were incubated with HRP-conjugated secondary antibodies for 1 h. The protein bands were captured using enhanced chemiluminescence (ECL) (Cell Signaling Technology) and the densitometry analysis was conducted using an Image J2x software (National Institute of Health, Bethesda, MD, USA).

### 4.8. RNA Extraction and Real-Time PCR

Total RNA from RAW264.7 macrophage cells or mouse lung tissues was extracted with Promega RNA extraction kit (Promega Corporation, Madison, WI, USA). RNA concentrations were determined spectrophotometrically and 2 µg of RNA was transcribed to cDNA using GoScript Reverse Transcription System (Promega Corporation). The polymerase reaction was performed using a 7500 Fast Real-Time PCR System (Applied Biosystems, Foster City, CA, USA) with the thermal cycle condition as follows: 50 °C for 2 min, and 40 cycles of amplification (95 °C for 15 s, 60 °C for 60 s, 72 °C for 1 min). The primer sequences used in this study are listed in Table S1. The expression level of each gene was calculated using the ΔΔCt method, and β-actin was used as an internal control.

### 4.9. Immunohistochemistry Analysis

The immunohistochemical analysis was performed on 4% formalin-embedded sections of lung tissues. The deparaffinized slices were incubated with primary antibodies against CD68 (cluster of differentiation 68, 1:50) and *O*-GlcNAc (1:100) at 4 °C overnight. After that, the sections were washed with PBS and incubated with biotinylated secondary antibodies for 1 h followed by the avidin-horseradish peroxidase (HRP) reagent for 30 min. Finally, the sections were stained with diaminobenzidine (DAB) and counterstained with hematoxylin. Images of lung sections were acquired using a DFC310 FX digital camera (Leica, Wetzlar, Germany) connected to a Leica DMI4000 B light microscope.

### 4.10. Statistical Analysis

Data were presented as mean ± SD. The statistical significance among multiple comparisons were determined using one-way ANOVA followed by Tukey for post hoc test. *p* values of < 0.05 were considered statistically significant. The statistical analysis was carried out with SPSS, version 20 (IBM, Armonk, NY, USA). 

## 5. Conclusions

We found that ACHO significantly inhibited LPS-induced inflammatory responses both in vitro and in vivo by suppressing the mRNA levels of pro-inflammatory cytokines including IL-6, IL-8, IL-1β, TNF-α and MCP-1. The potential molecular mechanism of anti-inflammation by ACHO led to the promotion of protein *O*-GlcNAcylation, which in turn inhibited the phosphorylation of p38 and Akt. This study suggests that pharmacological modulation of inflammatory reaction by AC oligosaccharides should be an effective approach for the prevention of diseases.

## Figures and Tables

**Table 1:** Relative abundance (%) of each oligosaccharide in *Antrodia cinnamomea* (AC) polysaccharides at 90 °C (ACHO) or 25 °C (ACCO).

Group	Hex_3_^1^	Hex_4_	Hex_5_	Hex_6_	Hex_7_	Hex_8_	Hex_9_	Hex_10_	Hex_11_
ACCO	27.25	20.76	17.49	11.62	10.94	5.11	3.63	1.68	1.52
ACHO	7.12	28.88	25.02	14.95	14.05	5.77	2.68	0.87	0.66

^1^ The relative abundance of each oligosaccharide was calculated on the bases of the ultra- performance liquid chromatography peak area. Hex represents hexose, and the subscript number indicates the degree of polymerization of each oligosaccharide.

**Table 2:** Monosaccharide composition of ACCO and ACHO.

Group	Composition	Ara ^1^	Glu ^2^	Fuc ^3^	Gal ^4^	Man ^5^
ACCO	mg/mL	0.06	0.23	0.01	1.21	3.49
	%	1.18	4.56	0.17	24.26	69.83
ACHO	mg/mL	0.02	0.22	0.03	1.22	3.51
	%	0.45	4.45	0.53	24.42	70.09

^1^ Ara, arabinose; ^2^ Glu, glucose; ^3^ Fuc, fucose; ^4^ Gal, galactose; ^5^ Man, mannose.

## Supplementary Materials

Supplementary materials are available online.

## References

[1] Yue P.Y., Wong Y.Y., Chan T.Y., Law C.K., Tsoi Y.K., Leung K.S. (2012). Review of biological and pharmacological activities of the endemic Taiwanese bitter medicinal mushroom, *Antrodia camphorata* (M. Zang et C.H. Su) Sh. H. Wu et al. (higher Basidiomycetes). Int. J. Med. Mushrooms.

[2] Lu M.C., El-Shazly M., Wu T.Y., Du Y.C., Chang T.T., Chen C.F., Hsu Y.M., Lai K.H., Chiu C.P., Chang F.R. (2013). Recent research and development of *Antrodia cinnamomea*. Pharmacol. Ther..

[3] Nakamura N., Hirakawa A., Gao J.J., Kakuda H., Shiro M., Komatsu Y., Sheu C.C., Hattori M. (2004). Five new maleic and succinic acid derivatives from the mycelium of *Antrodia camphorata* and their cytotoxic effects on LLC tumor cell line. J. Nat. Prod..

[4] Shie P.H., Wang S.Y., Lay H.L., Huang G.J. (2016). 4,7-Dimethoxy-5-methyl-1,3-benzodioxole from *Antrodia camphorata* inhibits LPS-induced inflammation via suppression of NF-kappaB and induction HO-1 in RAW264.7 cells. Int. Immunopharmacol..

[5] Yu C.-C., Chiang P.-C., Lu P.-H., Kuo M.-T., Wen W.-C., Chen P., Guh J.-H. (2012). Antroquinonol, a natural ubiquinone derivative, induces a cross talk between apoptosis, autophagy and senescence in human pancreatic carcinoma cells. J. Nutr. Biochem..

[6] Huang G.J., Deng J.S., Chen C.C., Huang C.J., Sung P.J., Huang S.S., Kuo Y.H. (2014). Methanol extract of *Antrodia camphorata* protects against lipopolysaccharide-induced acute lung injury by suppressing NF-kappaB and MAPK pathways in mice. J. Agric. Food Chem..

[7] Chen C.C., Liu Y.W., Ker Y.B., Wu Y.Y., Lai E.Y., Chyau C.C., Hseu T.H., Peng R.Y. (2007). Chemical characterization and anti-inflammatory effect of polysaccharides fractionated from submerge-cultured *Antrodia camphorata* mycelia. J. Agric. Food Chem..

[8] Cheng J.-J., Lu M.-K., Lin C.-Y., Chang C.-C. (2011). Characterization and functional elucidation of a fucosylated 1,6-alpha-d-mannogalactan polysaccharide from *Antrodia cinnamomea*. Carbohydr. Polym..

[9] Ho Y.-C., Lin M.-T., Duan K.-J., Chen Y.-S. (2008). The hepatoprotective activity against ethanol-induced cytotoxicity by aqueous extract of *Antrodia cinnamomea*. J. Chin. Inst. Eng..

[10] Wu Y.-Y., Chen C.-C., Chyau C.-C., Chung S.-Y., Liu Y.-W. (2007). Modulation of inflammation-related genes of polysaccharides fractionated from mycelia of medicinal basidiomycete *Antrodia camphorata*. Acta Pharmacol. Sin..

[11] Chyau C., Chen C., Wu Y., Liu Y., Peng R.Y. (2006). Antioxidative and anti-inflammatory activity of polysaccharides fractionated from submerge-cultured *Antrodia camphorata* mycelia. Free Radic. Res..

[12] Song A.-R., Qin D., Zhao C., Sun X.-L., Huang F., Kong C., Yang S. (2014). Immunomodulatory Effect of Polysaccharides Extracted from the Medicinal Mushroom *Antrodia camphorata* (Higher Basidiomycetes) in Specific Pathogen-Free Chickens. Int. J. Med. Mushrooms.

[13] Cheng J.-J., Chang C.-C., Chao C.-H., Lu M.-K. (2012). Characterization of fungal sulfated polysaccharides and their synergistic anticancer effects with doxorubicin. Carbohydr. Polym..

[14] Toress C.R., Hart G.W. (1984). Topography and Polypeptide Distribution of Terminal *N*-Acetylglucosamine Residues on the Surfaces of Intact Lymphocytes. J. Biol. Chem..

[15] Haltiwanger R.S., Holt G.D., Hart G.W. (1990). Enzymatic Addition of *O*-Glcnac to Nuclear and Cytoplasmic Proteins—Identification of a Uridine Diphospho-*N*-Acetylglucosamine-Peptide Beta-*N*-Acetylglucosaminyltransferase. J. Biol. Chem..

[16] Dong D.L., Hart G.W. (1994). Purification and characterization of an *O*-GlcNAc selective *N*-acetyl-beta-d-glucosaminidase from rat spleen cytosol. J. Biol. Chem..

[17] Bond M.R., Hanover J.A. (2013). *O*-GlcNAc cycling: A link between metabolism and chronic disease. Annu. Rev. Nutr..

[18] Naseem S., Parrino S.M., Buenten D.M., Konopka J.B. (2012). Novel roles for GlcNAc in cell signaling. Commun. Integr. Biol..

[19] Baudoin L., Issad T. (2015). *O*-GlcNAcylation and Inflammation: A Vast Territory to Explore. Front. Endocrinol..

[20] Li Y., Liu H., Xu Q.S., Du Y.G., Xu J. (2014). Chitosan oligosaccharides block LPS-induced *O*-GlcNAcylation of NF-kappaB and endothelial inflammatory response. Carbohydr. Polym..

[21] Not L.G., Brocks C.A., Vamhidy L., Marchase R.B., Chatham J.C. (2010). Increased O-linked beta-*N*-acetylglucosamine levels on proteins improves survival, reduces inflammation and organ damage 24 hours after trauma-hemorrhage in rats. Crit. Care Med..

[22] Rajapakse A.G., Ming X.F., Carvas J.M., Yang Z. (2009). O-linked beta-*N*-acetylglucosamine during hyperglycemia exerts both anti-inflammatory and pro-oxidative properties in the endothelial system. Oxid. Med. Cell. Longev..

[23] McCranie E.K., Bachmann B.O. (2014). Bioactive oligosaccharide natural products. Nat. Prod. Rep..

[24] Hilgers R.H., Xing D., Gong K., Chen Y.F., Chatham J.C., Oparil S. (2012). Acute *O*-GlcNAcylation prevents inflammation-induced vascular dysfunction. Am. J. Physiol. Heart Circ. Physiol..

[25] Siddhanta A.K., Goswami A.M., Ramavat B.K., Mody K.H., Mairh O.P. (2001). Water soluble polysaccharides of marine algal species of Ulva (Ulvales, Chlorophyta) of Indian waters. Indian J. Mar. Sci..

[26] Chen P.H., Weng Y.M., Yu Z.R., Koo M., Wang B.J. (2016). Extraction temperature affects the activities of antioxidation, carbohydrate-digestion enzymes, and angiotensin-converting enzyme of Pleurotus citrinopileatus extract. J. Food Drug Anal..

[27] Park J., Cha J.D., Choi K.M., Lee K.Y., Han K.M., Jang Y.S. (2017). Fucoidan inhibits LPS-induced inflammation in vitro and during the acute response in vivo. Int. Immunopharmacol..

[28] Li H., Xu H., Zhou Y., Zhang J., Long C., Li S., Chen S., Zhou J.M., Shao F. (2007). The phosphothreonine lyase activity of a bacterial type III effector family. Science.

[29] Manning B.D., Toker A. (2017). AKT/PKB Signaling: Navigating the Network. Cell.

[30] Chen Y.Y., Chou P.Y., Chien Y.C., Wu C.H., Wu T.S., Sheu M.J. (2012). Ethanol extracts of fruiting bodies of *Antrodia cinnamomea* exhibit anti-migration action in human adenocarcinoma CL1-0 cells through the MAPK and PI3K/AKT signaling pathways. Phytomedicine.

[31] Chen Y.Y., Liu F.C., Chou P.Y., Chien Y.C., Chang W.S., Huang G.J., Wu C.H., Sheu M.J. (2012). Ethanol extracts of fruiting bodies of *Antrodia cinnamomea* suppress CL1-5 human lung adenocarcinoma cells migration by inhibiting matrix metalloproteinase-2/9 through ERK, JNK, p38, and PI3K/Akt signaling pathways. Evid.-Based Complement. Altern. Med..

[32] Lu M.K., Lin T.Y., Chao C.H., Hu C.H., Hsu H.Y. (2017). Molecular mechanism of *Antrodia cinnamomea* sulfated polysaccharide on the suppression of lung cancer cell growth and migration via induction of transforming growth factor beta receptor degradation. Int. J. Biol. Macromol..

[33] Ozcan S., Andrali S.S., Cantrell J.E. (2010). Modulation of transcription factor function by *O*-GlcNAc modification. Biochim. Biophys. Acta.

[34] Goldberg H., Whiteside C., Fantus I.G. (2011). *O*-Linked beta-*N*-acetylglucosamine supports p38 MAPK activation by high glucose in glomerular mesangial cells. Am. J. Physiol. Endocrinol. Metab..

[35] Shi J., Gu J.H., Dai C.L., Gu J., Jin X., Sun J., Iqbal K., Liu F., Gong C.X. (2015). *O*-GlcNAcylation regulates ischemia-induced neuronal apoptosis through AKT signaling. Sci. Rep..

[36] Fulop N., Zhang Z., Marchase R.B., Chatham J.C. (2007). Glucosamine cardioprotection in perfused rat hearts associated with increased *O*-linked *N*-acetylglucosamine protein modification and altered p38 activation. Am. J. Physiol. Heart Circ. Physiol..

[37] Kang E.S., Han D., Park J., Kwak T.K., Oh M.A., Lee S.A., Choi S., Park Z.Y., Kim Y., Lee J.W. (2008). *O*-GlcNAc modulation at Akt1 Ser473 correlates with apoptosis of murine pancreatic beta cells. Exp. Cell Res..

[38] Vosseller K., Wells L., Lane M.D., Hart G.W. (2002). Elevated nucleocytoplasmic glycosylation by *O*-GlcNAc results in insulin resistance associated with defects in Akt activation in 3T3-L1 adipocytes. Proc. Natl. Acad. Sci. USA.

[39] Sevag M.G., Lackman D.B., Smolens J. (1938). The isolation of the components of streptococcal nucleoproteins in serologically active form. J. Biol. Chem..

[40] Mosmann T. (1983). Rapid colorimetric assay for cellular growth and survival: Application to proliferation and cytotoxicity assays. J. Immunol. Methods.

[41] Zachara N.E., Vosseller K., Hart G.W. (2011). Detection and Analysis of Proteins Modified by O-linked *N*-acetylglucosamine. Current Protocols in Protein Science.

