# Supplementary material for: Antrodia cinnamomea Oligosaccharides Suppress Lipopolysaccharide-Induced Inflammation through Promoting O-GlcNAcylation and Repressing p38/Akt Phosphorylation
Source: Molecules. 2017 Dec 26;23(1):51. doi: 10.3390/molecules23010051 (PMC5943963; doi:10.3390/molecules23010051)
Supplement: Supplementary file 1 [file molecules-23-00051-s001.pdf]

Supplementary information

- Figure S1and Figure S2 show the oligosaccharide composition.
- Figure S3 shows the monosaccharide composition.
- Table S1 displays the primers used in RT-PCR.
- All other text information descripts supplementary methods.

1. Oligosaccharide abundance of ACCO and ACHO by UPLC-MS analysis

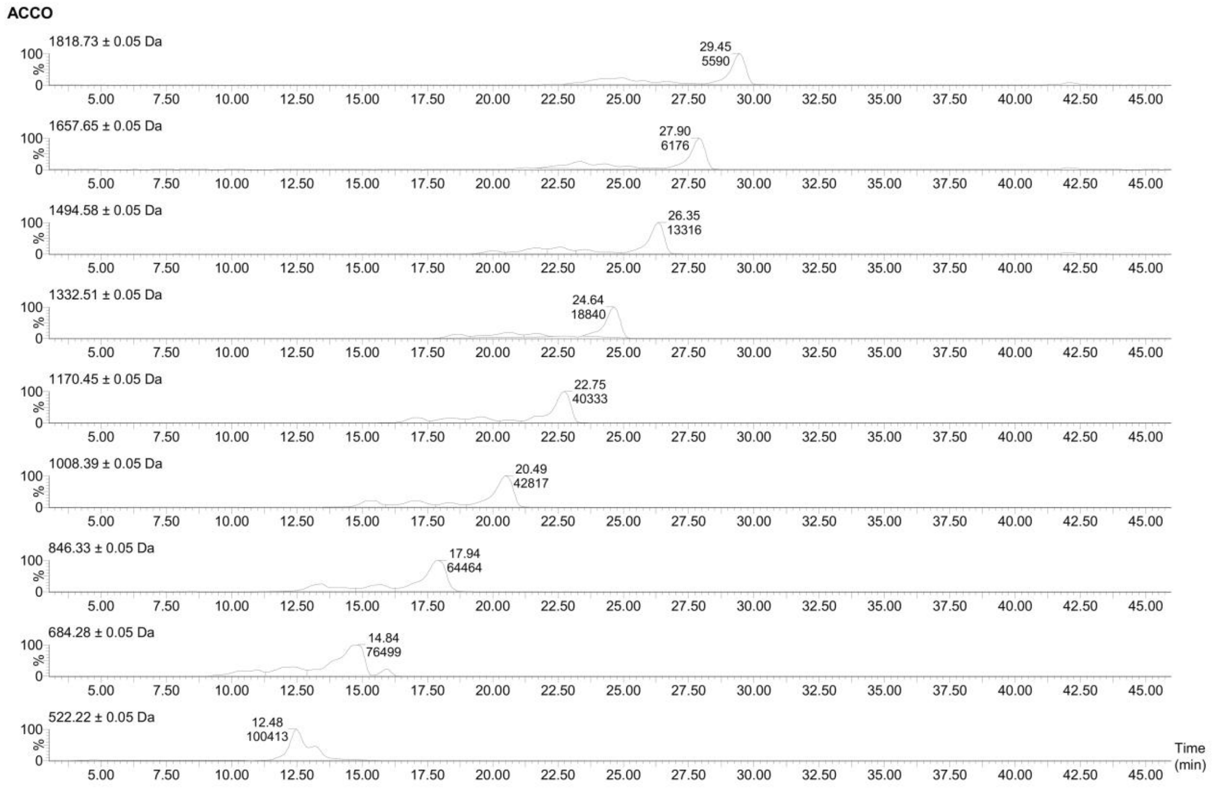

Figure S1. Ultra performance liquid chromatography of ACCO. The retention time and peak area of each oligosaccharide of ACCO were displayed.

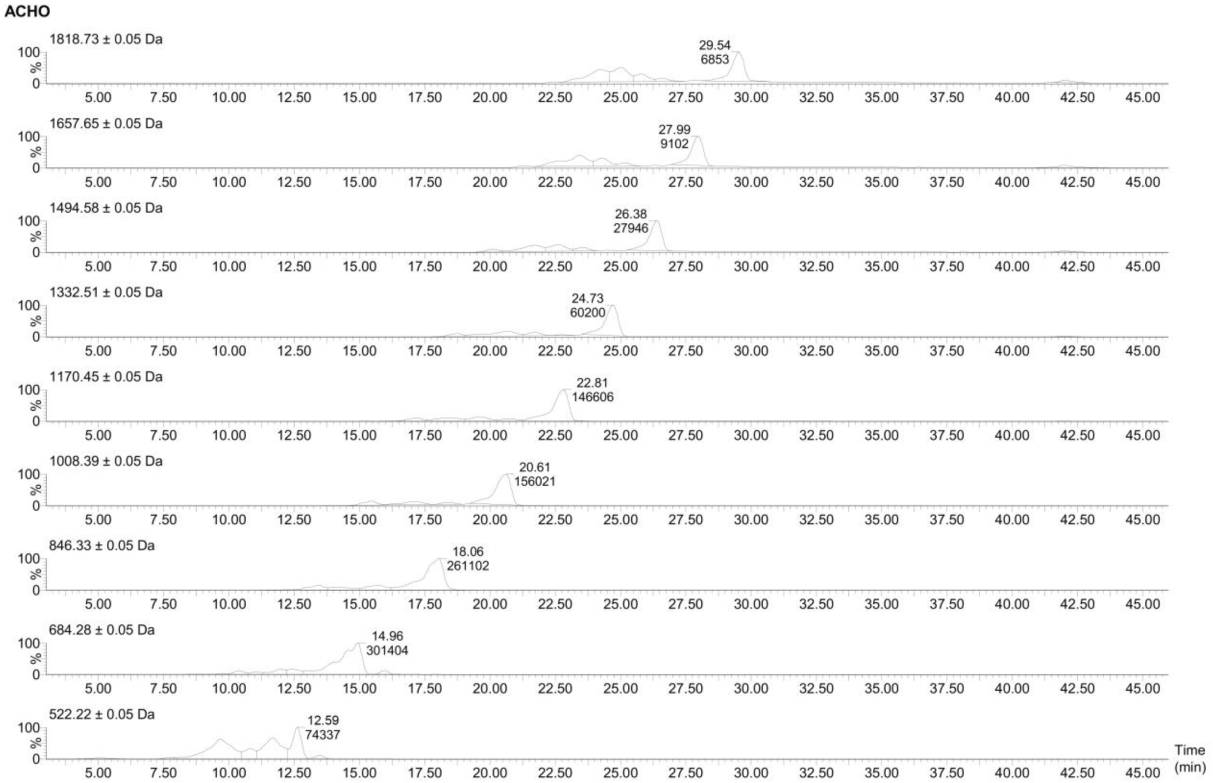

Figure S2. Ultra performance liquid chromatography of ACHO. The retention time and peak area of each oligosaccharide of ACHO were displayed.

2. PMP derivatization of monosaccharides

To prepare the PMP derivatives of monosaccharide standards, the following monosaccharides (10 mg for each) were dissolved in 5 ml of H2O and mixed well: D-(+)-arabinose, D-(+)-glucose, D-(+)-fucose, D-(+)-galactose, D-(+)-mannose. The derivatization reaction system was composed of 40 µl of standard mixture, 600 µl of NaOH (0.3 M) and 600 µl PMP (0.5 M) in methanol. After the incubation in water bath at 70 °C for 30 min, the reaction was stopped, cooled to room temperature and neutralized by addition of 600 µl of HCl (0.3 M). After extracted by 1 ml of chloroform, the aqueous layer was filtered with 0.22 µm membrane for capillary electrophoresis analysis. The PMP derivatives of hydrolyzed ACCO or ACHO were performed as above-mentioned.

3. Capillary electrophoresis analysis of PMP deratives

The PMP derivatives were separated using a P/ACE MDQ CE instrument (Beckman Coulter, Fullerton, CA, USA) with a UV detector at a wavelength of 245 nm. The analysis was performed on an Uncoated fused-silica capillary (Φ50 µm ×60 cm) at 26 °C. The mobile phase was a buffer solution of sodium borate (55 mM, pH 10.55). For each monosaccharide, the calibration factor (f_i) was calculated based on the peak area and molecular weight. The relative abundance of monosaccharide constituents was obtained by the ratio of f_i multiplied by their peak areas (A_i), as shown in the following:

$$\frac{n_i}{n_j} = \frac{f_i A_i}{f_j A_j}$$

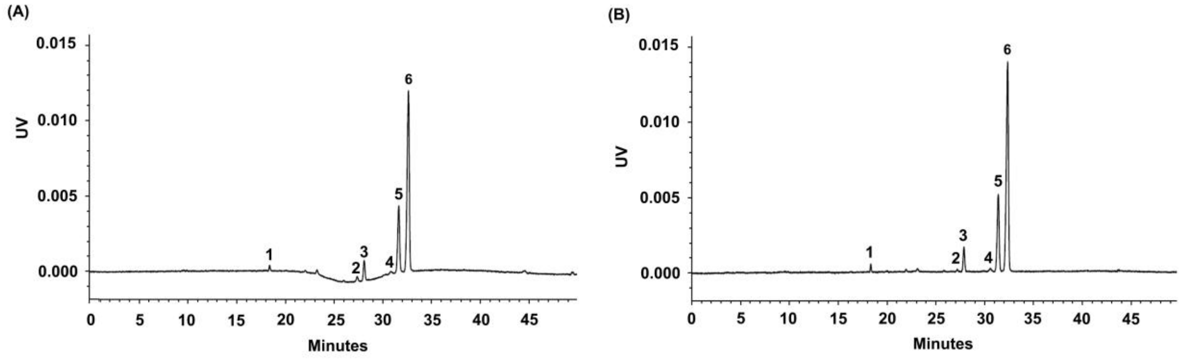

Figure S3. Composition analysis of ACHO and ACCO using capillary electrophoresis. A) Monosaccharide spectrum of ACCO; B) Monosaccharide spectrum of ACHO. 1, PMP; 2, arabinose; 3, glucose; 4, fucose; 5, galactose; 6, mannose.

4. Primers for real-time PCR

Table S1. The primer sequence used for RT-PCR

Primer name	Forward primer	Reverse primer
IL-1β	CGACAAAATACCTGTGGCCT	TTCTTTGGGTATTGCTTGGG
IL-6	GAAACCGCTATGAAGTTCCTCTCTG	TGTTGGGAGTGGTATCCTCTGTGA
IL-8	ATGGCTGGGATTCACCTCAA	AAGCCTCGCGACCATTCTT
TNF-α	AGGGTCTGGGCCATAGAACT	CCACCACGCTCTTCTGTCTAC
MCP-1	GGGATCATCTTGCTGGTGAA	AGGTCCCTGTCATGCTTCTG
β-actin	AGGTGACAGCATTGCTTCTG	GCTGCCTCAACACCTCAAC
